# SELF-NEGLECT IN OLDER ADULTS: CONVERSATIONS ABOUT SOCIAL CONNECTION, MEANING AND PURPOSE, AND INTERVENTIONS

**DOI:** 10.1093/geroni/igad104.2732

**Published:** 2023-12-21

**Authors:** Jason Burnett, Garielle Hoyumpa, Ashwin Kotwal, Sylvia Ayieko, Carla Perissinotto

**Affiliations:** University of Texas Health Science Center at Houston, Houston, Texas, United States; UTHealth, McGovern Medical School, Houston, Texas, United States; University of California San Francisco, San Francisco, California, United States; UTHealth, School of Public Health, Houston, Texas, United States; University of California San Francisco, San Francisco, California, United States

## Abstract

Social isolation and loneliness are pervasive health threatening conditions increasing the morbidity and mortality of older adults worldwide. Older adults experiencing self-neglect (SN) represent some of the most isolated and socially disconnected members of our communities living with high morbidity and mortality risks. We know very little about the interplay of SN, social isolation, loneliness, and related factors that could inform scalable social interventions for this population. We completed mixed methods structured conversations with N=10 older adults with Adult Protective Services validated SN, focusing on social isolation, loneliness, meaning and purpose, and intervention approaches. The average age was 77 years, 50% African American, 66% male, 83% lived alone with an average of 4 medical conditions. On standardized assessments, 66% reported high ability to engage in social activities, but all were socially isolated, with limited mobility. Eighty-three percent reported moderate to high loneliness, 50% with generalized anxiety and 17% with depression. Only 33% lacked meaning and purpose in their lives. The conversations suggest higher rates of depression. Loneliness was linked to living in limited life spaces and few meaningful and quality social interactions reflecting loss and personal impairments. Meaning and purpose was maintained through spirituality. All participants were willing to be engaged meaningfully and socially, in the future, by someone new to their life, in-person or via telephone, to increase social connection. These preliminary findings suggest that low-cost social connection interventions are acceptable options for improving the social lives and possibly the health of isolated and lonely older adults experiencing SN.

